# Metabolic engineering of *Caldicellulosiruptor bescii* yields increased hydrogen production from lignocellulosic biomass

**DOI:** 10.1186/1754-6834-6-85

**Published:** 2013-06-03

**Authors:** Minseok Cha, Daehwan Chung, James G Elkins, Adam M Guss, Janet Westpheling

**Affiliations:** 1 Department of Genetics, University of Georgia, Athens, GA 30602, USA; 2 Biosciences Division, Oak Ridge National Laboratory, Oak Ridge, TN 37831, USA; 3 The BioEnergy Science Center, Oak Ridge National Laboratory, Oak Ridge, TN 37831, USA

**Keywords:** *ldh*, Metabolic engineering, Switchgrass, Biohydrogen, *Caldicellulosiruptor*

## Abstract

**Background:**

Members of the anaerobic thermophilic bacterial genus *Caldicellulosiruptor* are emerging candidates for consolidated bioprocessing (CBP) because they are capable of efficiently growing on biomass without conventional pretreatment. *C. bescii* produces primarily lactate, acetate and hydrogen as fermentation products, and while some *Caldicellulosiruptor* strains produce small amounts of ethanol *C. bescii* does not, making it an attractive background to examine the effects of metabolic engineering. The recent development of methods for genetic manipulation has set the stage for rational engineering of this genus for improved biofuel production. Here, we report the first targeted gene deletion, the gene encoding lactate dehydrogenase (*ldh*), for metabolic engineering of a member of this genus.

**Results:**

A deletion of the *C. bescii* L-lactate dehydrogenase gene (*ldh*) was constructed on a non-replicating plasmid and introduced into the *C. bescii* chromosome by marker replacement. The resulting strain failed to produce detectable levels of lactate from cellobiose and maltose, instead increasing production of acetate and H_2_ by 21-34% relative to the wild type and *ΔpyrFA* parent strains. The same phenotype was observed on a real-world substrate – switchgrass (*Panicum virgatum*). Furthermore, the *ldh* deletion strain grew to a higher maximum optical density than the wild type on maltose and cellobiose, consistent with the prediction that the mutant would gain additional ATP with increased acetate production.

**Conclusions:**

Deletion of *ldh* in *C. bescii* is the first use of recently developed genetic methods for metabolic engineering of these bacteria. This deletion resulted in a redirection of electron flow from production of lactate to acetate and hydrogen. New capabilities in metabolic engineering combined with intrinsic utilization of lignocellulosic materials position these organisms to provide a new paradigm for consolidated bioprocessing of fuels and other products from biomass.

## Background

Fuel production from plant biomass offers the opportunity to generate energy from a sustainable feedstock, reduce dependence on petroleum, and reduce the negative environmental impact of increased CO_2_ emissions. The major obstacle in the use of lignocellulosic feedstocks is the recalcitrance of the biomass itself. Plants have evolved to resist deconstruction by microbes, and plant cell wall components such as cellulose, hemicellulose, and lignin play a major role in recalcitrance [1-3]. Industrial conversion of plant biomass to fuels currently relies on thermal and chemical treatment of biomass to remove hemicellulose and lignin, followed by enzymatic hydrolysis to solubilize the plant cell walls to generate a fermentable substrate for fuel-producing organisms [4-6]. However, these methods add cost, produce hydrolysates that are toxic to microorganisms [7] and are destructive to the sugars in the biomass [8]. An alternative approach is to use consolidated bioprocessing (CBP), in which the fermentative organism is also responsible for production of the biomass-solubilizing enzymes [9]. Members of the genus *Caldicellulosiruptor* are able to ferment all primary C5 and C6 sugars from plant biomass and are the most thermophilic cellulolytic bacteria known, with growth temperature optima between 78°C ~ 80°C [10]. They can also grow on and degrade biomass containing high lignin content as well as highly crystalline cellulose without conventional pretreatment [11-13], raising the possibility of further economic improvement of biofuel production from plant biomass by reducing or eliminating the pretreatment step.

While *Caldicellulosiruptor* species are attractive platforms for fuel and chemical production from plant biomass, the dearth of genetic tools for this genus has prevented rational strain development. Recent advances have enabled genetic transformation of *Caldicellulosiruptor bescii*[14], opening the possibility of metabolic engineering for improved biofuel production in this genus.

**Figure 1:**
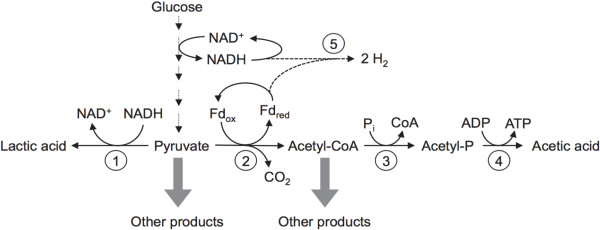
**A simplified version of predicted metabolic pathways for fermentation, glycolysis and electron transfer in *Caldicellulosiruptor bescii*.** (**1**) L-Lactate dehydrogenase; (**2**) Pyruvate-ferredoxin oxidoreductase; (**3**) Phosphotransacetylase; (**4**) Acetate kinase; (**5**) Bifurcating (reduced ferredoxin: NADH-dependent) hydrogenase. The thick grey arrows represent potential heterologous pathways that do not exist in *Caldicellulosiruptor* species but could be used for renewable fuel and chemical production.

During growth on glucose, *C. bescii* is predicted to utilize the Embden-Meyerhof glycolytic pathway to produce a combination of lactate and acetate + H_2_ + CO_2_ (see Figure 1 for a simplified pathway). As these are the only major fermentative products, pyruvate serves as the major metabolic branch point during fermentation, with carbon either being routed to lactate or acetyl-CoA and electrons being routed to lactate or H_2_. Thus, production of acetate is obligately coupled to H_2_ production to allow reoxidation of NADH and ferredoxin. While enteric bacteria such as *Enterobacter aerogenes*, *Enterobacter cloacae* and *Escherichia coli* produce 1 ~ 2 moles of H_2_ per mole of glucose [15,16], and *Clostridium* spp. can produce similar amounts [17-19], some hyperthermophiles such as *Thermococcales* spp., *Pyrococcus furiosus*, *Thermotogales* spp., and *Caldicellulosiruptor* spp. produce about 3 ~ 4 moles of H_2_ per mole of glucose [20-25]. Enterics typically utilize a formate-H_2_ lyase and *Clostridium* spp. use a ferredoxin-dependent hydrogenase to avoid the thermodynamically unfavorable formation of H_2_ from NADH, instead using other pathways such as ethanol or butanol production to reoxidize NADH. *Thermotoga* (and likely all the above-mentioned hyperthermophiles), on the other hand, use an electron bifurcating hydrogenase [26] to simultaneously oxidize NADH and ferredoxin to produce H_2_, using the thermodynamically favorable oxidation of ferredoxin to drive the unfavorable oxidation of NADH. This bifurcating hydrogenase allows a theoretical maximum yield of 4 moles of H_2_ per mole of glucose, whereas wild type *E. coli* has a theoretical maximum of 2 moles of H_2_ per mole of glucose. *C. bescii* encodes a putative bifurcating hydrogenase (Cbes1295-1299). This, combined with the native ability of *C. bescii* to catabolize plant biomass and the newly developed genetic transformation system, makes *C. bescii* a compelling platform to engineer high yield H_2_ production directly from plant biomass without conventional pretreatment.

We hypothesized that by developing the necessary tools to delete genes from the *C. bescii* chromosome [14,27,28], we would enable metabolic engineering to increase H_2_ production. Here, we demonstrate the utility of gene deletion in the pyruvate metabolic pathway for rational strain engineering of *C. bescii* while simultaneously creating a platform for further strain modification for advanced production of fuels and chemicals from renewable plant feedstocks.

## Results

### Deletion of lactate dehydrogenase (*ldh*) from the *C. bescii* chromosome

**Figure 2:**
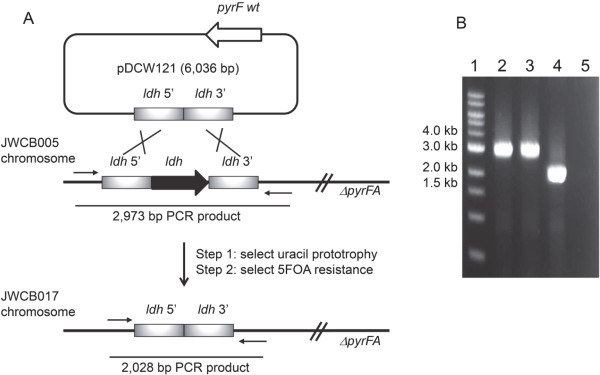
**Deletion of the *ldh *gene in *C. bescii*.** (**A**) A deletion cassette for the *ldh* gene was constructed in a non-replicating plasmid that contained a wild type copy of the *pyrF* gene, resulting in plasmid pDCW121. The cassette contained *ldh* 5′ and 3’ flanking DNA fragments. The plasmid was transformed into JWCB005, and uracil prototrophs were selected (resulting from plasmid insertion). Counter-selection with 5-FOA selected for strains that underwent a second recombination event, resulting in deletion of the marker and *ldh* to produce strain JWCB017. (**B**) Agarose gel showing PCR products amplified from the *ldh* locus in the wild type (lane 2), JWCB005 (*ΔpyrFA* parent strain*,* lane 3) and JWCB017 (*ΔpyrFA Δldh,* lane 4). Lane 1: 1 kb DNA ladder; Lane 5: no template PCR control. Expected bands: wild type *ldh* locus – 3 kb; *ldh* deletion – 2.0 kb.

We recently reported a method for DNA transformation and marker replacement in *Caldicellulosiruptor bescii* based on uracil prototrophic selection [14,27,28]. *C. bescii* strain JWCB005 (*ΔpyrFA, ura*^*-*^*/5-FOA*^*R*^) contains a deletion of the *pyrFA* locus making the strain a uracil auxotroph resistant to 5-fluoroorotic acid (5-FOA) [27], allowing the use of *pyrF* as both a selectable and counter-selectable marker (Figure 2A). A deletion of the L-lactate dehydrogenase gene (Cbes1918) was constructed by fusing the 5′ and 3′ flanking regions of the *ldh* gene and cloning the fused product into a non-replicating plasmid vector, resulting in plasmid pDCW121. This vector also contains the wild type *pyrF* allele under the transcriptional control of a ribosomal protein gene promoter (Cbes2105, 30S ribosomal protein S30EA), allowing both positive (uracil prototrophy) and negative (5-FOA sensitivity) selection. Plasmid pDCW121 was transformed into *C. bescii* JWCB005 selecting uracil prototrophy resulting from plasmid recombination into the targeted region, followed by counter-selecting 5-FOA resistance (resulting from plasmid excision). The resulting strain, JWCB017, contained a deletion of the *ldh* wild type gene in the chromosome. To confirm the *ldh* deletion in JWCB017, the region of the *ldh* locus was amplified by PCR using primers outside of the plasmid regions of homology used to construct the deletion (Figure 2B). The wild type and the *ΔpyrFA* strain (JWCB005) gave the same expected 3.0 kb bands, while PCR from JWCB017 resulted in the smaller 2.0 kb band, as predicted. The PCR product was also sequenced to verify that the deletion in the chromosome was the same as that constructed on the plasmid.

### Deletion of *ldh* eliminates lactate production and increases acetate and H_2_ production

**Figure 3:**
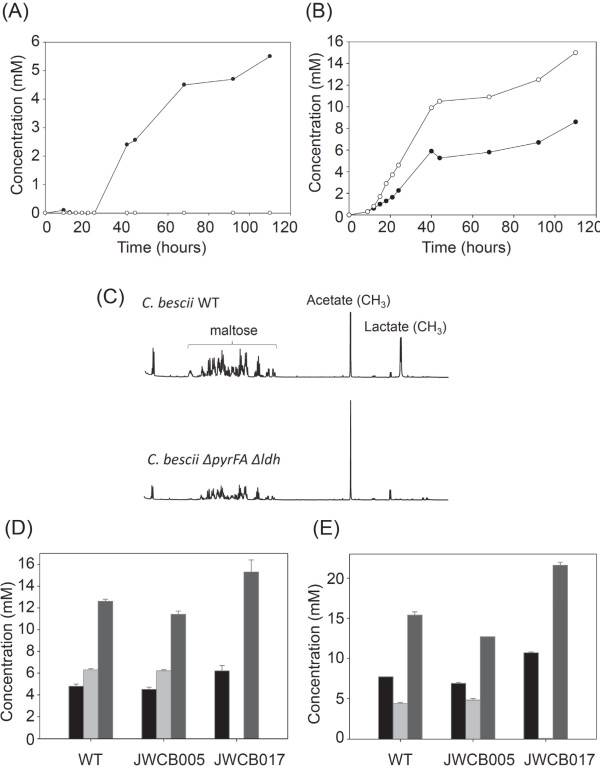
**Fermentation products by *C. bescii *mutant strains.** (**A**) Lactic acid and (**B**) acetic acid production were measured during growth on 0.5% maltose by the parent strain (JWCB005 *ΔpyrFA*; filled circles) and JWCB017 (*ΔpyrFA Δldh*; open circles). (**C**) Production of lactic and acetic acids by wild type and JWCB017 were further measured by NMR analysis after 48 hours incubation. (**D**) End products of *C. bescii* wild-type and mutant strains were measured by HPLC on cellobiose after 30 hours incubation, and (**E**) switchgrass after 120 hours incubation. Acetate, Black; Lactate, Light gray; Hydrogen, Dark gray.

Cbes1918 is the only predicted lactate dehydrogenase gene encoded in the *C. bescii* genome. To confirm that this gene is solely responsible for the production of lactate in *C. bescii*, wild type, JWCB005 and JWCB017 were grown on 0.5% maltose, and fermentation products were analyzed by high-performance liquid chromatography (HPLC) (Figure 3AB) and nuclear magnetic resonance (NMR) analysis (Figure 3C). No lactate was detected in the mutant by either method, as compared to approximately 5.0 mM lactate from the wild-type and parental strains.

To compare the production of lactate, acetate and hydrogen, *C. bescii* wild-type and mutant strains were grown in LOD medium [29] with soluble cellodextrans (cellobiose) or plant biomass (switchgrass) as carbon source. When grown on 0.5% cellobiose for 30 hours, JWCB017 showed 29% and 21% more acetate production and 37% and 34% more hydrogen production than wild type and parent strains, respectively (Figure 3D). Cells grown for 120 hours on LOD medium supplemented with 0.5% switchgrass as the sole carbon source showed a similar profile to that on cellobiose, with the *Δldh* strain producing 38% and 40% more acetate and 55% and 70% more hydrogen than wild-type and parent strains (Figure 3E).

### Growth yield increases upon deletion of *ldh*

**Figure 4:**
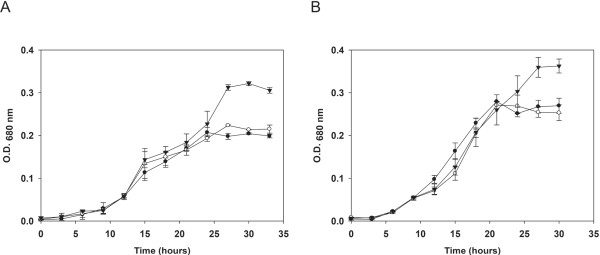
**Growth (O.D.**_**680 nm**_**) comparison of wild-type and mutant strains.** (**A**) 0.5% of maltose and (**B**) 0.5% cellobiose as the carbon source; filled circles, Wild-type; open circles, *ΔpyrFA* (JWCB005); filled triangles, *ΔpyrFA Δldh* (JWCB017). Error bars based on three biologically independent experiments.

Growth of JWCB017 was compared to the wild type and parental strains in defined media [29] supplemented with either 0.5% maltose or 0.5% cellobiose. While growth of the *ΔpyrFA* parent strain on both maltose (Figure 4A) and cellobiose (Figure 4B) was indistinguishable from the wild type [29], the JWCB017 mutant strain reached a 34-53% higher final optical density than the wild type and parent. Interestingly, while the growth rate was comparable, the exponential growth phase of JWCB017 was extended resulting in higher cell densities.

## Discussion

We have built upon recent advances in the genetic manipulation of *Caldicellulosiruptor*[14,27,28] to delete the gene encoding lactate dehydrogenase. While the wild type strain produced roughly equimolar amounts of acetate and lactate, the JWCB017 mutant strain no longer produced lactate, instead rerouting carbon and electron flux to acetate and H_2_, respectively. JWCB001 (~1.8 mol/mol of glucose) and JWCB005 (~1.7 mol/mol of glucose) appeared a bit lower in hydrogen yield than reported values for *C. saccharolyticus* (~2.5 mol/mol of glucose) [22]. However, in this report, *C. saccharolyticus* was grown in culture media with added yeast extract, which improves yields [22]. JWCB017 (~3.4 mol/mol of glucose) was more than that reported for *C. saccharolyticus*. Yield and titer of acetate and H_2_ were increased in the *C. bescii ldh* deletion strain from both model soluble substrates and real-world plant biomass. A similar approach has been applied to other thermophilic biomass-degrading bacteria, including xylanolytic *Thermoanaerobacterium saccharolyticum* and cellulolytic *Clostridium thermocellum*[30-32], with the goal of increasing ethanol production.

Members of the genus *Caldicellulosiuptor* offer special advantages for biomass conversion to products of interest in that they are hyperthermophiles with optimal growth temperatures between 70°C ~ 80°C and they are capable of using biomass without conventional pretreatment.

Interestingly, deletion of *ldh* resulted in a higher cell yield and longer exponential growth phase relative to the wild type. The increase in cell density is likely caused by an increase in acetate production, which should increase ATP production per glucose via acetate kinase providing more energy for biosynthesis and growth. The evolutionary pressures that selected for maintenance of *ldh* are not clear, though it may be related to the partial pressure of H_2_ found in the environment. Further, the molecular mechanism by which *C. bescii* switches from production of acetate + H_2_ to lactate is unknown. *C. thermocellum* encodes a lactate dehydrogenase that is allosterically activated by fructose-1,6-bisphosphate [33], such that lactate is only produced when the rate of substrate uptake exceeds glycolytic flux. It would be interesting to examine whether *C. bescii* utilizes a similar mechanism for flux control at the pyruvate node of glycolysis. Independent of mechanism, the fact that *C. bescii* JWCB017 grows to a higher density without an obvious effect on growth rate suggests that further engineered strains may be able to compete well with the wild type strain and thrive in an industrial setting.

Recent progress in genetic tool development opened the possibility of more advanced metabolic engineering strategies to increase the utility of *C. bescii* for industrial applications. In addition to the construction of gene deletions, this will enable gene insertion into the chromosome (so called gene knock-ins), simplifying the process of heterologous gene expression by eliminating the need for plasmid maintenance and increasing the number of genes that can be stably expressed. Thus, we have created a new platform for rational strain design for lignocellulosic bioconversion, enabling future efforts to increase the titer of H_2_, express heterologous pathways for production of liquid fuels and chemicals, increase robustness, and improve upon the native ability of *Caldicellulosiruptor* species to deconstruct and convert biomass without conventional pretreatment.

Characterization of the JWCB017 mutant also sheds light on the basic physiology of *C. bescii*, which will inform future metabolic modeling and engineering efforts. For instance, this strain has now been engineered to produce only acetate and H_2_ from sugars, providing further evidence that *C. bescii* uses a bifurcating hydrogenase to funnel all the electrons to H_2_. Examination of the *C. bescii* genome sequence reveals that glycolysis likely yields NADH from glyceraldehyde-3-phosphate oxidation, based on the presence of glyceraldehyde-3-phosphate dehydrogenase (Cbes1406) and lack of glyceraldehyde-3-phosphate: ferredoxin oxidoreductase. Conversion of pyruvate to acetyl-CoA, on the other hand, presumably reduces ferredoxin, based on the presence of pyruvate: ferredoxin oxidoreductase (Cbes0874-0877) and the lack of pyruvate dehydrogenase and pyruvate-formate lyase. Because H_2_ evolution from NADH is thermodynamically unfavorable except in extremely low H_2_ partial pressures [34], this implies that the favorable production of H_2_ from ferredoxin drives the unfavorable NADH-dependent H_2_ production. Further genetic modification will increase our understanding of metabolic flux in *C. bescii*, allowing better metabolic models and further informing metabolic engineering efforts.

## Conclusions

Here we show the first application of recently developed genetic methods for metabolic engineering of a member of the genus *Caldicellulosiruptor*. The method for creating a deletion of the *ldh* gene in the *C. bescii* chromosome was efficient enough to allow targeted marker replacement using non-replicating plasmids. The resulting mutant grew to a higher cell density and produced more hydrogen than the wild-type strain. Using the tools developed here, *C. bescii* JWCB017 will serve as a platform for additional rational strain engineering for production of fuels and chemicals from lignocellulosic feedstocks.

## Methods

### Strains, growth conditions and molecular techniques

A spontaneous mutant containing a deletion within the *pyrFA* locus of *C. bescii*, JWCB005 [27,28], was used in this study to select transformants. *C. bescii* strains were grown in modified DSMZ516 medium or LOD (low osmolality defined growth medium) [29] containing 0.5% maltose as carbon source, final pH 7.0. Liquid cultures were grown from a 0.5% inoculum or a single colony and incubated at 75°C in anaerobic culture bottles degassed with five cycles of vacuum and argon. A solid medium was prepared by mixing an equal volume of liquid medium at a 2× concentration with the same volume of (wt/vol) agar, 3.6% (Difco, Sparks, MD) that had been previously autoclaved. Both solutions were maintained at 70°C and poured into petri dishes immediately after mixing. A series of dilutions of this culture were mixed with 4 ml of soft top agar (1.5% of agar) and poured across the top of the solid agar medium. The plates were degassed with five cycles of vacuum and argon and incubated at 75°C for 4 days in anaerobic jars. *E. coli* DH5α was used to prepare plasmid DNA. Cells were grown in LB broth supplemented with apramycin (50 μg/ml). Plasmid DNA was isolated using a Qiagen Mini-prep Kit (QIAGEN inc., Valenica, CA). A complete list of strains, plasmids, and primers used in this study is shown in Tables 1 and 2.

**Table 1:** **Plasmids and *C. bescii *strains (JWCB) used in this study**

**Strains and Plasmids**	**Description and/or relevant characteristics**	**Source or reference**
pDCW88	Non-replicating plasmid in *C. bescii*	[28]
pDCW121	*ldh* knock-out plasmid	This study
JWCB001	*C. bescii* wild-type DSM 6725	[35]
JWCB005	DSM 6725 *ΔpyrFA*	[27]
JWCB017	DSM 6725 *ΔpyrFA Δldh*	This study

**Table 2:** Oligonucleotides used in this study

**Primer**	**Sequence 5’- 3’**
DC081	TCCAATGATCGAAGTTAGGCTGGT
DC348	GAATTCTCTGACGCTCAGTGGAACGAA
DC349	GAAAACAAATGGGCTTGGGAGGATAGGAGGCTGT
DC350	TGGGCTTGGGAGGATAGGAGGCTGTCTAAAAACAA
DC351	TGCCAAGATATGAAATGAGAACT
DC356	CGTCTCATCTGTGCATATGGACAGTTATAATCCCAAAAGGAGGATTGGATCC

### Construction of pDCW121

To construct a plasmid for deletion of the *ldh* gene (Cbes1918), three cloning steps including overlapping polymerase chain reactions were used. All PCR amplifications were performed using *Pfu* Turbo DNA polymerase (Agilent Tech., Santa Clara, CA). A 1,009 bp fragment containing a KpnI site upstream of the *ldh* gene was amplified using primers DC348 and DC349. A 1,011 bp fragment containing an EcoRI site downstream of *ldh*, was amplified using primers DC350 and DC351. The two fragments were joined by overlapping PCR using primers DC348 and DC351 to generate a 2,020 bp product that was cloned into pDCW88 [28] using the Kpnl and EcoRI sites. The resulting plasmid, pDCW121, was transformed into *E. coli* DH5α by an electrotransformation via a single electric pulse (1.8 kV, 25 μF and 200 Ω) in a pre-chilled 1 mm cuvette using a Bio-Rad gene Pulser (Bio-Rad, Hercules, CA). Transformants were selected on LB solid medium containing apramycin (50 μg/ml final).

### Competent cells, transformation and mutant selection in *C. bescii*

To prepare competent cells, a 50 ml culture of JWCB005 was grown in LOD minimal medium at 75°C for 18 hours (to mid exponential phase) and 25 ml of the culture was used to inoculate a 500 ml culture of LOD (low osmolarity defined growth medium) supplemented with 40 μM uracil and a mixture of 19 amino acids (5% inoculum, v/v) [29]. The 500 ml culture was incubated at 75°C for 5 hours and cooled to room temperature for 1 hr. Cells were harvested by centrifugation (6000 × g, 20 min) at 25°C and washed three times with 50 ml of pre-chilled 10% sucrose. After the third wash, the cell pellet was resuspended in 50 μl of pre-chilled 10% sucrose in a microcentrifuge tube and stored at −80°C until needed. Before transformation, plasmids from *E. coli* cells were methylated *in vitro* with *C. bescii* methyltransferase (M.CbeI, [14]) and methylated plasmid DNAs (0.5-1.0 μg) were added to the competent cells, gently mixed and incubated for 10 minutes in ice. Electrotransformation of the cell/DNA mixture was performed via single electric pulse (1.8 kV, 25 μF and 350 Ω) in a pre-chilled 1 mm cuvette using a Bio-Rad gene Pulser (Bio-Rad, Hercules, CA). After pulsing, cells were inoculated into 10 ml of LOC medium (low osmolarity complex growth medium, [29]) and incubated for 4 hours at 75°C. 100 μl of the culture was transferred into 20 ml of defined medium without uracil. After 18 hours incubation at 75°C, cells were harvested by centrifugation (at 6000 × g for 20 min) and resuspended in 1 ml of 1× basal salts. 100 microliters of the cell suspension was plated onto solid defined media with 40 μM uracil and 8 mM 5-FOA (5- fluoroorotic acid monohydrate).

### Analytical techniques for determining fermentation end products

Batch fermentations were conducted in stoppered 125 ml serum bottles containing 50 ml LOD medium with 5 g/l maltose, cellobiose or switchgrass. Cultures of JWCB005 and JWCB017 were supplemented with 40 μM uracil. Triplicate bottles were inoculated with a fresh 2% (v/v) inoculum and incubated at 75°C without shaking. Total cell dry weight (CDW) was determined by concentrating 25 ml of each culture on dried, preweighed 47 mm Supor membrane filters (0.45, Pall Corp., Ann Arbor, MI) and washed with 10 ml of _dd_H_2_O. Cell retentates were dried for 16 hours at 85°C and weighed on an analytical balance. Culture supernatants were analyzed via HPLC using a Waters Breeze 2 system (Waters Chromatography, Milford, MA) operated under isocratic conditions at 0.6 ml/min with 5 mM H_2_SO_4_ as a mobile phase. Analytes were separated on an Aminex HPX-87H column (Bio-Rad Laboratories, Hercules, CA) at 60°C and monitored via refractive index (RI) using a Waters 2414 RI detector. Total peak areas were integrated using Waters Breeze 2 software and compared against peak areas and retention times of known standards for each analyte of interest. H_2_ was measured using an Agilent Technologies 6850 Series II Gas Chromatograph equipped with a thermal conductivity detector at 190°C with a N_2_ reference flow and a HP-PLOT U Column (30 m * 0.32 mm). To measure organic acid production, Nuclear magnetic resonance (NMR) analysis was performed. One-dimensional 1H-NMR spectra were recorded at 298 K with a Varian Inova-NMR operating at 600 MHz for 1H and equipped with a 5-mm NMR cold probe. Samples (500 μL) of cell free culture media were mixed with 150 μL of D_2_O as internal lock and immediately analyzed. 128 scans were recorded for each sample using a pre-saturation method to suppress the water resonance. The amounts of the most abundant components in the samples were calculated by integration of the proton signals in the spectra. The data were normalized to the amount of acetic acid in each sample.

### Biomass preparation

Air-dried switchgrass (*Panicum virgatum*, Alamo variety) was reduced to 60 mesh using a Wiley Mini-Mill (Thomas Scientific, Swedesboro, NJ, USA). The ground switchgrass was subjected to a hot water treatment similar to that described by Yang et. al. [12] however the biomass was boiled in distilled H_2_O (2% w/v) for 1 hour rather than treating overnight at 75°C. The switchgrass was then washed and dried overnight at 50°C before dispensing into serum bottles as previously described [12].

## Abbreviations

ldh: L-lactate dehydrogenase; CBP: Consolidated bioprocessing; 5-FOA: 5-Fluoroorotic aicd; HPLC: High-performance liquid chromatography; NMR: Nuclear magnetic resonance; LOD: Low osmolarity defined growth medium; LOC: Low osmolarity complex growth medium; CDW: Cell dry weight; RI: Refractive index; GC: Gas chromatography.

## Competing interests

The authors declare that they have no competing interests.

## Authors’ contributions

MC and DC designed and carried out the genetic and growth experiments, analyzed results and participated in the writing of the manuscript. JGE and AMG designed and carried out the analysis of fermentation products and participated in writing the manuscript. JW participated in design of the study, coordination of the work, and writing of the manuscript. All authors read and approved the final manuscript.

## References

[B1] Himmel ME Ding SY Johnson DK Adney WS Nimlos MR Brady JW Foust TD Biomass recalcitrance: engineering plants and enzymes for biofuels production Science 2007 315 804 807 10.1126/science.1137016 17289988

[B2] Wilson DB Three microbial strategies for plant cell wall degradation Ann N Y Acad Sci 2008 1125 289 297 10.1196/annals.1419.026 18378599

[B3] McCann MC Carpita NC Designing the deconstruction of plant cell walls Curr Opin Plant Biol 2008 11 314 320 10.1016/j.pbi.2008.04.001 18486537

[B4] Zhang YH Ding SY Mielenz JR Cui JB Elander RT Laser M Himmel ME McMillan JR Lynd LR Fractionating recalcitrant lignocellulose at modest reaction conditions Biotechnol Bioeng 2007 97 214 223 10.1002/bit.21386 17318910

[B5] Negro MJ Manzanares P Ballesteros I Oliva JM Cabanas A Ballesteros M Hydrothermal pretreatment conditions to enhance ethanol production from poplar biomass Appl Biochem Biotechnol 2003 105–108 87 100 12721477 10.1385/abab:105:1-3:87

[B6] Wyman CE What is (and is not) vital to advancing cellulosic ethanol Trends Biotechnol 2007 25 153 157 10.1016/j.tibtech.2007.02.009 17320227

[B7] Klinke HB Thomsen AB Ahring BK Inhibition of ethanol-producing yeast and bacteria by degradation products produced during pre-treatment of biomass Appl Microbiol Biotechnol 2004 66 10 26 10.1007/s00253-004-1642-2 15300416

[B8] Barakat A Monlau F Steyer JP Carrere H Effect of lignin-derived and furan compounds found in lignocellulosic hydrolysates on biomethane production Bioresour Technol 2012 104 90 99 22100239 10.1016/j.biortech.2011.10.060

[B9] Lynd LR Weimer PJ van Zyl WH Pretorius IS Microbial cellulose utilization: fundamentals and biotechnology Microbiol Mol Biol Rev 2002 66 506 577 10.1128/MMBR.66.3.506-577.2002 12209002 PMC120791

[B10] Hamilton-Brehm SD Mosher JJ Vishnivetskaya T Podar M Carroll S Allman S Phelps TJ Keller M Elkins JG *Caldicellulosiruptor obsidiansis* sp. nov., an anaerobic, extremely thermophilic, cellulolytic bacterium isolated from Obsidian Pool, Yellowstone National Park Appl Environ Microbiol 2010 76 1014 1020 10.1128/AEM.01903-09 20023107 PMC2820981

[B11] Blumer-Schuette SE Kataeva I Westpheling J Adams MW Kelly RM Extremely thermophilic microorganisms for biomass conversion: status and prospects Curr Opin Biotechnol 2008 19 210 217 10.1016/j.copbio.2008.04.007 18524567

[B12] Yang SJ Kataeva I Hamilton-Brehm SD Engle NL Tschaplinski TJ Doeppke C Davis M Westpheling J Adams MW Efficient degradation of lignocellulosic plant biomass, without pretreatment, by the thermophilic anaerobe “*Anaerocellum thermophilum*” DSM 6725 Appl Environ Microbiol 2009 75 4762 4769 10.1128/AEM.00236-09 19465524 PMC2708433

[B13] Blumer-Schuette SE Giannone RJ Zurawski JV Ozdemir I Ma Q Yin Y Xu Y Kataeva I Poole FL 2nd Adams MW *Caldicellulosiruptor* core and pangenomes reveal determinants for noncellulosomal thermophilic deconstruction of plant biomass J Bacteriol 2012 194 4015 4028 10.1128/JB.00266-12 22636774 PMC3416521

[B14] Chung D Farkas J Huddleston JR Olivar E Westpheling J Methylation by a unique alpha-class N4-cytosine methyltransferase is required for DNA transformation of *caldicellulosiruptor bescii* DSM6725 PLoS One 2012 7 e43844 10.1371/journal.pone.0043844 22928042 PMC3425538

[B15] Tanisho S Kamiya N Wakao N Hydrogen evolution of *Enterobacter aerogenes* depending on culture pH: mechanism of hydrogen evolution from NADH by means of membrane-bound hydrogenase Biochim Biophys Acta 1989 973 1 6 10.1016/S0005-2728(89)80393-7 2643990

[B16] Yoshida A Nishimura T Kawaguchi H Inui M Yukawa H Enhanced hydrogen production from glucose using *ldh*- and *frd*-inactivated *Escherichia coli* strains Appl Microbiol Biotechnol 2006 73 67 72 10.1007/s00253-006-0456-9 16683133

[B17] Liu X Zhu Y Yang ST Construction and characterization of ack deleted mutant of *Clostridium tyrobutyricum* for enhanced butyric acid and hydrogen production Biotechnol Prog 2006 22 1265 1275 17022663 10.1021/bp060082g

[B18] Collet C Girbal L Peringer P Schwitzguebel JP Soucaille P Metabolism of lactose by *Clostridium thermolacticum* growing in continuous culture Arch Microbiol 2006 185 331 339 10.1007/s00203-006-0098-4 16508746

[B19] Chin HL Chen ZS Chou CP Fedbatch operation using *Clostridium acetobutylicum* suspension culture as biocatalyst for enhancing hydrogen production Biotechnol Prog 2003 19 383 388 10.1021/bp0200604 12675576

[B20] Kanai T Imanaka H Nakajima A Uwamori K Omori Y Fukui T Atomi H Imanaka T Continuous hydrogen production by the hyperthermophilic archaeon, *Thermococcus kodakaraensis* KOD1 J Biotechnol 2005 116 271 282 10.1016/j.jbiotec.2004.11.002 15707688

[B21] Kadar Z de Vrije T van Noorden GE Budde MA Szengyel Z Reczey K Claassen PA Yields from glucose, xylose, and paper sludge hydrolysate during hydrogen production by the extreme thermophile *Caldicellulosiruptor saccharolyticus* Appl Biochem Biotechnol 2004 114 497 508 10.1385/ABAB:114:1-3:497 15054273

[B22] Kadar Z de Vrije T Mars AE Budde MA Lai MH Dijkema C de Waard P Claassen PA Glycolytic pathway and hydrogen yield studies of the extreme thermophile *Caldicellulosiruptor saccharolyticus* Appl Microbiol Biotechnol 2007 74 1358 1367 10.1007/s00253-006-0783-x 17216445

[B23] Schroder C Selig M Schonheit P Glucose fermentation to acetate, CO_2_ and H_2_ in the anaerobic hyperthermophilic eubacterium *thermotoga-maritima* - involvement of the embden-meyerhof pathway Arch Microbiol 1994 161 460 470

[B24] Willquist K Zeidan AA van Niel EW Physiological characteristics of the extreme thermophile *Caldicellulosiruptor saccharolyticus*: an efficient hydrogen cell factory Microb Cell Fact 2010 9 89 10.1186/1475-2859-9-89 21092203 PMC3003633

[B25] Schicho RN Ma K Adams MW Kelly RM Bioenergetics of sulfur reduction in the hyperthermophilic archaeon *Pyrococcus furiosus* J Bacteriol 1993 175 1823 1830 8449888 10.1128/jb.175.6.1823-1830.1993 PMC203983

[B26] Schut GJ Adams MW The iron-hydrogenase of *Thermotoga maritima* utilizes ferredoxin and NADH synergistically: a new perspective on anaerobic hydrogen production J Bacteriol 2009 191 4451 4457 10.1128/JB.01582-08 19411328 PMC2698477

[B27] Chung D Cha M Farkas J Westpheling J Construction of a stable replicating shuttle vector for *caldicellulosiruptor* species: Use for extending genetic methodologies to other members of this genus PLoS One 2013 8 e62881 10.1371/journal.pone.0062881 23658781 PMC3643907

[B28] Chung D Farkas J Westpheling J Overcoming restriction as a barrier to DNA transformation in *Caldicellulosiruptor* species results in efficient marker replacement with non-replicating plasmid vectors Biotech biofuels 2013 6 82 10.1186/1754-6834-6-82 PMC3679861 23714229

[B29] Farkas J Chung D Cha M Copeland J Grayeski P Westpheling J Improved growth media and culture techniques for genetic analysis and assessment of biomass utilization by *Caldicellulosiruptor bescii* J Ind Microbiol Biotechnol 2013 40 41 49 10.1007/s10295-012-1202-1 23149625 PMC4290016

[B30] Shaw AJ Podkaminer KK Desai SG Bardsley JS Rogers SR Thorne PG Hogsett DA Lynd LR Metabolic engineering of a thermophilic bacterium to produce ethanol at high yield Proc Natl Acad Sci USA 2008 105 13769 13774 10.1073/pnas.0801266105 18779592 PMC2544529

[B31] Argyros DA Tripathi SA Barrett TF Rogers SR Feinberg LF Olson DG Foden JM Miller BB Lynd LR Hogsett DA Caiazza NC High ethanol titers from cellulose by using metabolically engineered thermophilic, anaerobic microbes Appl Environ Microbiol 2011 77 8288 8294 10.1128/AEM.00646-11 21965408 PMC3233045

[B32] Tripathi SA Olson DG Argyros DA Miller BB Barrett TF Murphy DM McCool JD Warner AK Rajgarhia VB Lynd LR Development of *pyrF*-based genetic system for targeted gene deletion in *Clostridium thermocellum* and creation of a pta mutant Appl Environ Microbiol 2010 76 6591 6599 10.1128/AEM.01484-10 20693441 PMC2950449

[B33] Ozkan M Yilmaz EI Lynd LR Ozcengiz G Cloning and expression of the *Clostridium thermocellum* L-lactate dehydrogenase gene in *Escherichia coli* and enzyme characterization Can J Microbiol 2004 50 845 851 10.1139/w04-071 15644899

[B34] Thauer RK Jungermann K Decker K Energy conversion in chemotrophic anaerobic bacteria Bacteriol Rev 1971 41 100 180 860983 10.1128/br.41.1.100-180.1977 PMC413997

[B35] Svetlichnyi VA Svetlichnaya TP Chernykh NA Zavarzin GA Anaerocellum-thermophilum Gen-Nov Sp-Nov - an extremely thermophilic cellulolytic eubacterium isolated from Hot-springs in the valley of geysers Microbiol 1990 59 598 604

